# Bidirectional Effects between Parenting and Aggressive Child Behavior in the Context of a Preventive Intervention

**DOI:** 10.1007/s10802-016-0211-3

**Published:** 2016-10-27

**Authors:** Lysanne W. te Brinke, Maja Deković, Sabine E. M. J. Stoltz, Antonius H. N. Cillessen

**Affiliations:** 1 Department of Child and Adolescent Studies, Utrecht University, 3584 CS Utrecht, the Netherlands; 2 Behavioral Science Institute, Radboud University, 6500 HE Nijmegen, the Netherlands

**Keywords:** Aggressive behavior, Perceived parenting, Bidirectionality, Intervention, Correlated change

## Abstract

Over time, developmental theories and empirical studies have gradually started to adopt a bidirectional viewpoint. The area of intervention research is, however, lagging behind in this respect. This longitudinal study examined whether bidirectional associations between (changes in) parenting and (changes in) aggressive child behavior over time differed in three conditions: a child intervention condition, a child + parent intervention condition and a control condition. Participants were 267 children (74 % boys, 26 % girls) with elevated levels of aggression, their mothers and their teachers. Reactive aggression, proactive aggression and perceived parenting were measured at four measurement times from pretest to one-year after intervention termination. Results showed that associations between aggressive child behavior and perceived parenting are different in an intervention context, compared to a general developmental context. Aggressive behavior and perceived parenting were unrelated over time for children who did not receive an intervention. In an intervention context, however, decreases in aggressive child behavior were related to increases in perceived positive parenting and decreases in perceived overreactivity. These findings underscore the importance of addressing child-driven processes in interventions aimed at children, but also in interventions aimed at both children and their parents.

According to socialization theories, the association between childhood aggression and parenting can best be described as bidirectional (O’Connor 2002). As Bell (1968) stated decades ago, a unidirectional view of parent-child socialization overlooks the child as a potential part of the environment for the parent. Following Bell’s review, developmental theories and empirical studies gradually started to adopt a bidirectional viewpoint (Pardini 2008). There is a specific research area, however, that is lagging behind in this respect: intervention research. In intervention studies, it is often assumed that it is the change in parenting that leads to changes in child behavior. However, bidirectional effects also are likely to occur in an intervention context because, for example, parents are instructed to respond less coercively. In addition, direct aggression is particularly likely to affect parenting because of its visible and troubling nature (De Haan et al. 2013). Therefore, the goal of this study was to examine whether bidirectional associations differ in an intervention context as compared to a general developmental context.

## Bidirectional Effects in a Developmental Context

Patterson’s coercion model provides a classic conceptualization of bidirectional influences between parenting and aggressive behavior. According to Patterson, children who show aggressive behavior elicit aversive reactions, such as negative discipline, from their parents (Patterson et al. 1984). In reaction to these parental control attempts, children try to resist their parents by increasing the intensity of problem behavior. Over time, this can lead to a reciprocal pattern of negative parent-child interactions. Longitudinal studies show that negative parenting practices (e.g., ineffective discipline) are positively related to aggressive child behavior (Snyder et al. 2005) and that positive parenting practices (e.g., sensitivity, warmth) are negatively related to aggressive child behavior (Boeldt et al. 2012). Besides effects from parenting to child behavior, child-driven effects also have been demonstrated. Aggressive behavior was found to predict higher levels of perceived parental psychological control (Murray et al. 2013). Furthermore, adolescent girls’ externalizing problem behavior was found to predict decreases in perceived parental support and control (Huh et al. 2006). However, the reverse influence from perceived parenting to externalizing problem behavior was not found (Huh et al. 2006).

Bidirectional effects are also reported in the literature. For example, results from a longitudinal genetically informed study supported a bidirectional association between child antisocial behavior and parental negativity (Larsson et al. 2008). However, other studies suggested that parenting and child behavior do not influence each other over time. For example, Vuchinich et al. (1992) found that adolescent antisocial behavior and parental discipline techniques were not related over time. Thus, inconsistent results have been found regarding the direction of effects.

## Bidirectional Effects in an Intervention Context

To our knowledge, only one study examined bidirectionality in an intervention context. Shaffer et al. (2013) examined bidirectional effects between aggressive child behavior and parenting in a sample of 6–11 year old children in multi-modal treatment for ODD or CD directed at both parents and children. Overall, bidirectional effects were smaller than the temporal stability of parenting and child behavior. However, Shaffer et al. (2013) did not include a control condition and thus could not examine differences between a general developmental and an intervention context. Moreover, they only assessed negative parenting constructs, whereas research suggests that (bidirectional) associations might differ depending on the construct that is assessed. In a longitudinal study that examined bidirectional influences in a clinic-referred sample of 7–17 year old boys, bidirectional associations only were observed for specific parenting behaviors (Burke et al. 2008). Although a reciprocal relationship was observed between timid parental discipline and increases in ODD symptoms, findings for other parenting behaviors (e.g., parental involvement, supervision) were more supportive of child effects (Burke et al. 2008).

## Current Study

The aim of this study was to examine whether the association between (changes in) parenting and (changes in) aggressive child behavior over time differed in three conditions: a child intervention condition, a child + parent intervention condition and a control condition. The inclusion of three conditions enabled us to examine whether bidirectional associations differ between an intervention context and a general developmental context. We included both negative and positive parenting constructs. Because the literature regarding bidirectional effects is inconsistent, we cannot propose a strong hypothesis for the control condition, which reflects normative development of children with elevated levels of aggression. In the child intervention condition, we expected a decrease in aggression. In turn, parents may find it easier to show more positive and less negative parenting behavior. Therefore, we expected that in the child intervention condition, child-driven effects might be stronger than parent-driven effects. In the child + parent intervention condition, parenting skills were also targeted. Therefore, in this condition we expected to find parent-driven effects in addition to child-driven effects.

The association between changes in parenting and changes in aggression was examined with two different approaches. We first examined differences between the three conditions in correlated change with the multigroup bivariate Latent Growth Modeling (LGM) method. Testing correlated change is an important extension on earlier studies that examined bidirectional relations. Whereas these earlier studies mainly investigated time-specific associations between variables (e.g., correlations or cross-lagged effects), correlated change examines the association between two developmental trajectories. This enabled us to examine whether (changes in) parenting and (changes in) aggression influence each other over time. However, the direction of causality and timing of effects cannot be determined with the correlated change approach. Therefore, multi-group bivariate LGM was supplemented with another time-based approach: cross-lagged modeling. The use of cross-lagged models enabled us to test the direction and timing of effects. These models were also examined with multigroup analyses, to test whether the effects differed between the three conditions.

We measured both mother- and teacher reported aggression because earlier studies reported differences in the strength of effects between informants of child behavior. Specifically, Pardini et al. (2008) found that the influence of child behavior on changes in parenting was stronger when child conduct problems were measured by parents rather than teachers. While this could be explained by the fact that conduct problems exhibited at home can influence parents’ behaviors more than those exhibited at school, it could also be due to shared informant variance, as parents reported on both conduct problems and parenting. In the current study, child reports of parenting were used to reduce shared method bias.

The intervention in this study is a social-cognitive intervention for children at elementary schools with elevated levels of aggression. Previous studies found that the intervention reduces aggression and improves social cognitive functioning and self-esteem (Stoltz et al. 2013a, b). The present study extended the literature on relationships between aggressive child behavior and parenting by examining differences in bidirectional associations between a general developmental and an intervention context. Examining these associations in an intervention context is consistent with needs identified in the child treatment literature to examine treatment processes and mechanisms of change (Kazdin and Nock 2003). While treatment processes and mechanisms of change are well documented for parent-directed interventions, this is not the case for interventions that target both parents and children.

## Method

### Design

In this randomized controlled trial, 48 elementary schools in two urban regions in the Netherlands were randomly assigned to one of three groups: a child intervention condition, a child + parent intervention condition or a control condition. Each school participated in the intervention conditions, as well as in the control condition, but in a different order. In this way, schools were more willing to participate in the control condition. Moreover, treatment condition was randomized in such a way to ensure that intervention effects could not be due to school factors. There were four measurement times: prior to the beginning of the child intervention (T1), at the end of child intervention and before the start of the parent intervention (T2, 11 weeks after T1), a half-year follow-up (T3) and a 1-year follow-up (T4). The study was approved by the Dutch Central Committee on Research Involving Human Subjects.

### Participants

**Fig. 1:**
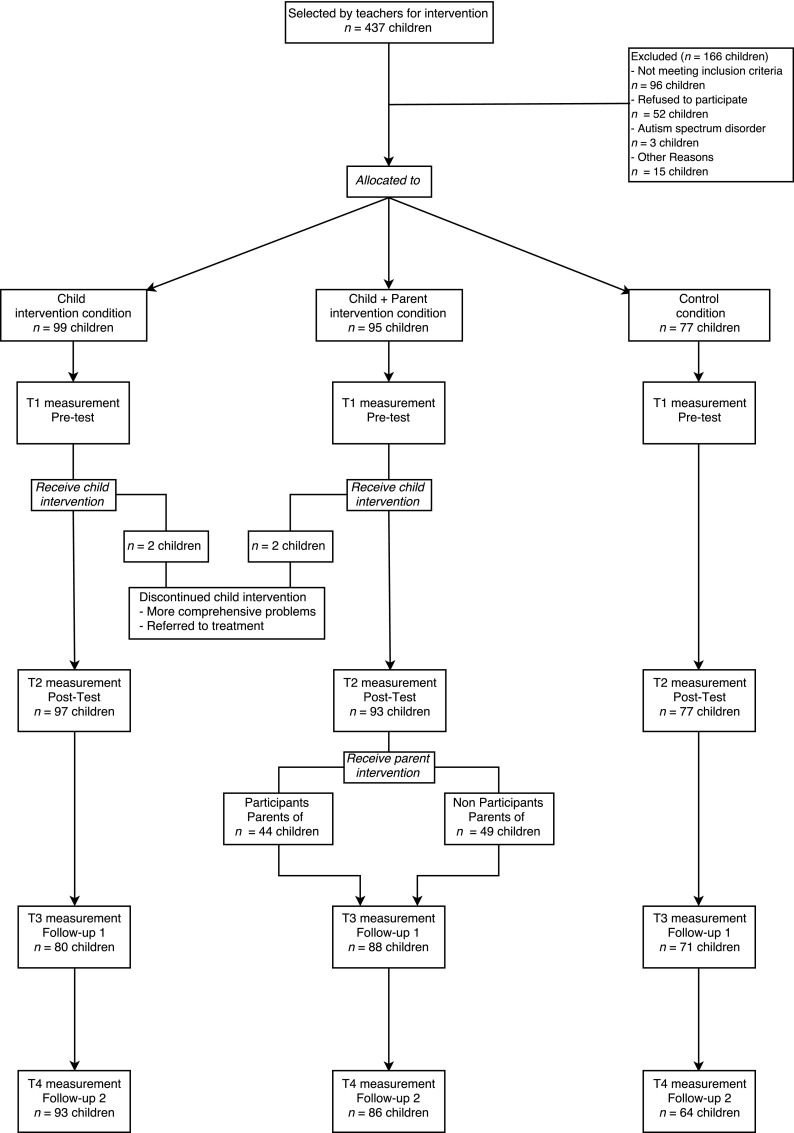
Flow chart of randomization design

Parents of all children in the fourth grades of participating elementary schools received a general information letter and a consent form to give permission to their child’s teacher to fill out the Teacher Report Form (TRF 6–18; Achenbach and Rescorla 2001). Teachers nominated the children with the highest levels of externalizing behavior in their classroom (the top 30 %) and completed the externalizing scale of the TRF for them. Based on the teacher nominations, researchers initially selected 437 children for participation. Next, from this sample, researchers selected children who had a threshold clinical level of externalizing behavior (T-score > 60) and obtained informed consent for further participation from their primary caregivers. Figure 1 presents the flow chart of participants. Children were excluded if: (a) they did not meet the inclusion criterion of a TRF Externalizing T-score > 60, (b) participated in other forms of youth care, (c) were diagnosed with autism spectrum disorder, or (d) their parents did not give consent. All parents of the participating children gave informed consent.

The final sample consisted of 267 children (74 % boys, 26 % girls), their mothers and their teachers. Of these children, 97 were assigned to the child intervention condition, 93 to the child + parent intervention condition and 77 to the control condition.^1^ Children in the control condition did not receive an intervention, and therefore this condition reflects normative development. It is important to note, however, that these children also showed elevated levels of aggression. At the first measurement time, children were between 8 and 12 years old (*M* = 9.54, *SD* = 0.61). In total, 37 % of the children were immigrants. Consistent with other studies in the Netherlands (Eichelsheim et al. 2010), a child was considered immigrant if either the child or one of the parents was born in another country than the Netherlands. No significant differences were found among the three conditions at pretest on ethnicity, gender of the child, child age, or levels of reactive and proactive aggression. Child measures were collected at school by trained research assistants. Mothers and teachers received questionnaires by mail.

The sample of the current study was also used in three earlier papers (Stoltz et al. 2013a, b; Stoltz et al. 2015). In previous reports, however, only the child intervention and control condition (Stoltz et al. 2013a, b) or child intervention and child + parent intervention conditions were included (Stoltz et al. 2015). In the current study, all three conditions were included, because we aimed to study differences between intervention and general developmental contexts. Moreover, the purpose of the present study is clearly different. Whereas earlier studies mainly focused on the effectiveness of the intervention, the present study focuses on bidirectional associations between (changes in) parenting and (changes in) aggression.

### Intervention

Children in the child intervention participated in Stay Cool Kids (Stoltz et al. 2013b), a school-based social cognitive intervention designed to reduce aggressive behavior of highly aggressive children at elementary schools. Stay Cool Kids consists of eight 45-min individual training sessions at school. In the first phase of the intervention, Stay Cool Kids trainers investigate the child’s specific needs and competences. In the second phase, trainers perform exercises with the child, focused on changing self-perception, social cognitions, and anger management to reduce aggressive behavior. Parents and teachers receive information after each training session about the content of the session and are asked to practice newly learned skills with their child. Previous studies found that the Stay Cool Kids child intervention is effective in reducing aggressive behavior and improving social cognitive functioning and self-esteem (Stoltz et al. 2013a, b).

The parent intervention is an individually delivered parent managed training, in line with the child intervention (similar exercises and terminology). The aim of the parent intervention is to improve parenting skills that affect parent-child interactions. After the last session of the child intervention, parents are invited by the trainer to take over their coaching role while being supervised by the same trainer. The intervention is tailor made and adapted to specific needs of the parents in relation to their child’s behavior. Stay Cool Kids trainers and parents decide together which parenting skills they will focus on, for example: positive reinforcement, extinction and ignoring, rule-setting, positive problem solving, communication, and involvement. Parents receive a manual with information and coaching exercises, and a DVD with positive examples. Trainers have weekly individual contact with parents by e-mail or telephone. In an earlier study, it was found that participation in the parent intervention resulted in increased maternal involvement and seemed to interrupt the development of more aggressive behavior and less appropriate parenting skills for children in highest need (Stoltz et al. 2015). Participation in the parent intervention, however, did not directly affect aggressive child behavior.

### Measures

At all four measurement times, mothers and teachers reported on aggressive child behavior and children reported on perceived parenting. Both parent and teacher reports of aggressive behavior were used because research suggests that bidirectional effects may differ by informant (Pardini et al. 2008). Child reports of parenting were used to reduce shared method variance and because this is consistent with the view that the impact of parenting on child adjustment is mediated by how children perceive their parents’ behavior (Neiderhiser et al. 1998). Perceptions of both positive and negative parenting were included, because researchers have suggested that parenting can be classified in two fundamental domains: supportive/positive parenting and inconsistent/negative parenting (Barber et al. 2005).

#### Child Aggressive Behavior

The Externalizing subscale of the Teacher Report Form (TRF) age 6–18 was used as the screening measure (Achenbach and Rescorla 2001). Reactive and proactive aggression were measured with the Teacher Rating of Aggression (TRA; Dodge and Coie 1987). Items for both reactive aggression (3 items, i.e., “When this child has been teased or threatened, he or she gets angry easily and strikes back”) and proactive aggression (3 items, i.e., “This child uses force to dominate peers”) were rated on a 5-point scale (1 = *never*, 5 = *always*). Mothers rated reactive and proactive aggression with an adapted parent version of the TRA. Over measurement times, Cronbach’s α ranged from 0.72 to 0.80 for mother reported reactive aggression, from 0.75 to 0.80 for mother reported proactive aggression, from 0.82 to 0.89 for teacher reported reactive aggression, and from 0.78 to 0.88 for teacher reported proactive aggression. Pearson correlations between mother and teacher reports ranged from 0.04 to 0.17 for reactive aggression and from 0.14 to 0.19 for proactive aggression. Thus, correlations between teacher and parent reported aggression within the same measurement time were low, as reported in the literature (Achenbach et al. 1987).

#### Perceived Positive Parenting

Perceived positive parenting was assessed with the child form of the Alabama Parenting Questionnaire (APQ; Elgar et al. 2007; Shelton et al. 1996). Children reported on the level of perceived positive parenting that they received from their mothers. The subscale positive parenting consisted of six items (i.e., “My mother tells me that I am doing a good job”) that were rated on a 5-point scale (1 = *never*, 5 = *always*). A mean score was constructed, with a high score indicating a high level of perceived positive parenting. Cronbach’s α ranged from 0.79 to 0.94 across measurement times.

#### Perceived Overreactivity

Reformulated items of the Parenting Scale (Arnold et al. 1993) were used to assess children’s perceived overreactive parenting. The overreactivity scale initially consisted of eight items to be answered on a 7-point Likert scale. However, reliability analyses showed that one item (“When I misbehave, my mother spanks, slaps or hits me”) negatively influenced Cronbach’s α. Therefore, this item was excluded from the analyses. Thus, the final scale consisted of seven items (i.e., “When I misbehave, my mother uses bad language or curses”). A mean score was constructed with a high score indicating a high level of perceived overreactive parenting. Cronbach’s α ranged from ﻿0.64 to 0﻿.76 across measurement times.

### Data Analyses

An examination of missing data revealed that mothers of six children and teachers of four other children did not report on aggressive behavior on all four occasions. Moreover, some participants dropped-out on another measurement time (see Fig. 1). Attrition analyses were performed, in which the sample with complete data was compared with a sample of participants with incomplete data. The sample of participants with incomplete data consisted of more children in the control condition (54 %), compared to the sample with complete data (26 %), χ^2^ (2) = 8.92, *p* = 0.012. In addition, teacher reported reactive aggression at baseline was higher (*M* = 3.84, *SD* = 0.87) in the sample of participants with incomplete data, compared to the sample with complete data (*M* = 3.68, *SD* = 0.98), *t*(266) = −1.25, *p* = 0.048. There were no significant differences between the complete and incomplete samples regarding mother reported aggression, teacher reported proactive aggression, age, ethnicity or gender. Little’s Missing Completely At Random (MCAR) test produced a normed χ^2^ (χ^2^/*df*) of 1.07, which indicates that data was missing at random (Little 1988). Because multiple imputation has been recommended as a technique to handle missing data when data is MCAR (Baraldi and Enders 2010), we used Multiple Imputation in Mplus (Muthén and Muthén 2012).

The analyses were run with Mplus 7.11. Model fit indices were evaluated with the χ^2^ likelihood ratio statistic, the root mean square error of approximation (RMSEA), and the comparative fit index (CFI). RMSEA values less than 0.08 and CFI values greater than 0.90 indicate acceptable model fit (Kline 2005). Full Information Maximum Likelihood was used.

First, trajectories of the six constructs (mother reported reactive and proactive aggression, teacher reported reactive and proactive aggression, perceived positive parenting, perceived overreactivity) were identified and modeled with univariate latent growth models (LGMs). We examined a linear model with an *intercept factor* (with factor loadings of four observed variables, corresponding to four measurement times, set at 1) and a *slope factor* (with the factor loadings set at 0, 0.24, 0.84 and 1.44 to account for unequal intervals between measurement times). If a linear model did not fit the data, a model in which the slope factor loading was estimated freely for one of the measurement times was estimated.

To determine the association between changes in reactive/proactive aggression and changes in perceived parenting, differences between the three groups in correlated change were examined with the multigroup bivariate LGM method, in which the best-fitting univariate LGMs were combined. Eight bivariate LGMs were estimated, each with a different combination of perceived parenting and aggression constructs. Growth parameters of aggressive behavior and perceived parenting were estimated simultaneously and were allowed to correlate. Wald tests were used to compare significant slope-slope correlations between groups.

**Fig. 2:**
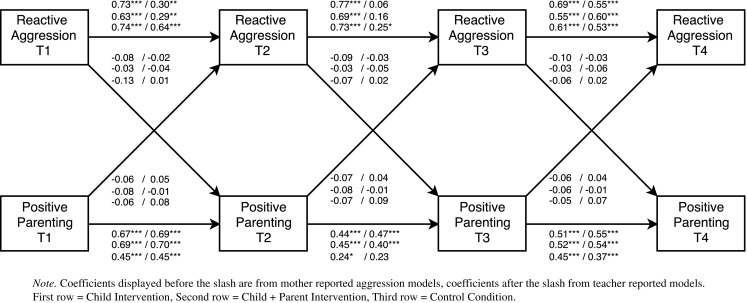
Cross-lagged path model examining bidirectional effects between reactive aggression and perceived positive parenting. All coefficients are standardized

**Fig. 3:**
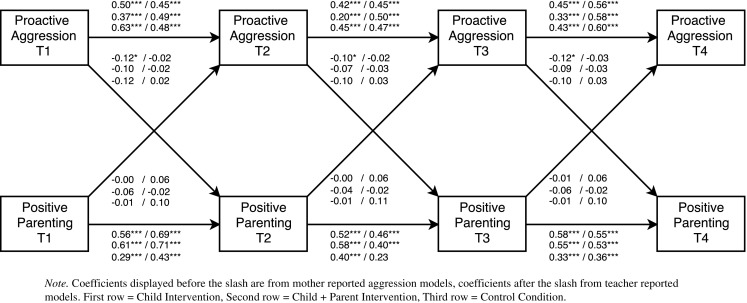
Cross-lagged path model examining bidirectional effects between proactive aggression and perceived positive parenting. All coefficients are standardized

**Fig. 4:**
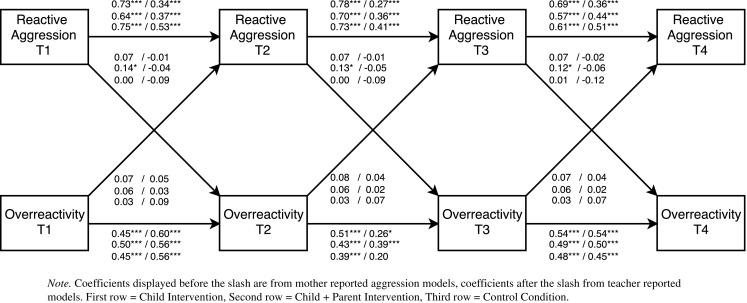
Cross-lagged path model examining bidirectional effects between reactive aggression and perceived overreactivity. All coefficients are standardized

**Fig. 5:**
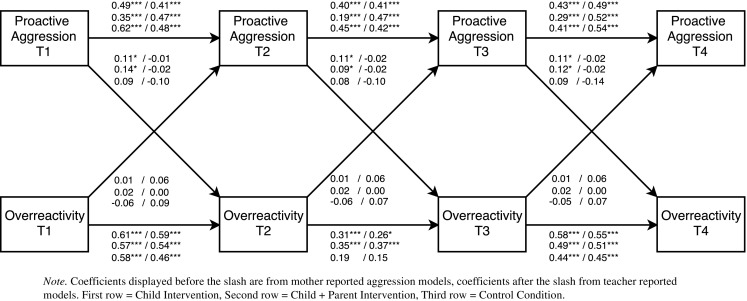
Cross-lagged path model examining bidirectional effects between proactive aggression and perceived overreactivity. All coefficients are standardized

To determine the direction and timing of bidirectional effects, multi-group cross-lagged models were examined. In total, eight separate cross-lagged models were tested. Each model included the autoregressive paths (predicting a construct from its prior level) and the cross-lagged paths (connecting child behavior and parenting across adjacent time points). Further, residual correlations between aggressive behavior and parenting at each time point were added to control for initial correlations between constructs. If the fit of the initial model was not satisfactory, additional stability paths from pretest to follow-up 1 (T1 to T3) and from post-test to follow-up 2 (T2 to T4) were added. These paths were added only if they improved model fit and did not change the stability and cross-lagged paths (see Figs. 2, 3, 4, and 5 2). After a model was established that fitted the data, we first tested whether the stability paths of parenting an aggressive behavior could be constrained to be equal over time. Next, we tested whether the cross-lagged paths from aggressive behavior to parenting and the cross-lagged paths from parenting to aggressive behavior could be constrained to be equal over time. The fit of nested models was compared with χ^2^ difference tests.^3^ If constraining paths to be equal over time did not result in a significant decrease in model fit, this indicates that there is time-invariance. Group differences in significant cross-lagged paths were examined with Wald Tests.

## Results

### Univariate LGMs

Means and standard deviations for all variables are presented in Table 1. Fit statistics are included in Table 2. For mother reported reactive aggression, a linear growth model did not fit the data, χ^2^(15) = 32.11, *p* = 0.006, CFI = 0.936, RMSEA =0.113. A model in which the slope factor loading of the posttest (T2) was freely estimated fitted the data significantly better, Δχ^2^(1) = 16.17, *p* < 0.001. The estimated model showed that the decrease in mother reported reactive aggression was steeper from T1 to T2 than later on. For teacher reported reactive aggression, a linear growth model also did not fit the data, χ^2^(15) = 35.07, *p* = 0.002, CFI = 0.840, RMSEA =0.123. In this case, a model in which the slope of the second follow-up (T4) was freely estimated fitted the data significantly better, Δχ^2^ (1) = 13.09, *p* < 0.001. The estimated model showed that the decrease in teacher reported reactive aggression was less steep from T3 to T4 than immediately after the intervention. For the other four constructs, a linear model showed good model fit (Table 2).

**Table 1:** Descriptive statistics: means and SD

Outcome	Child intervention (*n* = 97)				Child + Parent intervention (*n* = 93)				Control condition (*n* = 77)			
	Pre	Post	Fu1	Fu2	Pre	Post	Fu1	Fu2	Pre	Post	Fu1	Fu2
Reactive aggression M	3.07 (0.98)	2.67 (0.90)	2.68 (0.97)	2.55 (0.93)	2.95 (0.87)	2.65 (0.82)	2.61 (0.82)	2.52 (0.96)	2.91 (0.93)	2.73 (0.86)	2.64 (0.85)	2.42 (0.86)
Proactive aggression M	1.55 (0.68)	1.43 (0.59)	1.31 (0.62)	1.31 (0.56)	1.56 (0.70)	1.38 (0.48)	1.41 (0.63)	1.34 (0.47)	1.59 (0.73)	1.45 (0.56)	1.50 (0.57)	1.47 (0.60)
Reactive aggression T	3.87 (0.90)	3.41 (0.88)	3.00 (1.06)	2.94 (1.10)	3.81 (0.84)	3.34 (0.98)	2.99 (1.13)	3.02 (1.27)	3.74 (0.95)	3.56 (1.00)	3.22 (1.07)	2.91 (1.14)
Proactive aggression T	2.52 (0.96)	2.04 (0.97)	1.98 (1.00)	1.85 (0.94)	2.40 (0.94)	2.10 (1.07)	1.90 (0.98)	1.86 (0.99)	2.16 (0.89)	2.12 (0.94)	1.91 (0.94)	1.78 (0.97)
Positive parenting C	4.00 (0.79)	4.07 (0.81)	4.14 (0.75)	4.07 (0.67)	4.11 (0.81)	4.11 (0.80)	4.20 (0.68)	4.16 (0.69)	4.19 (0.65)	4.20 (0.78)	4.20 (0.78)	4.23 (0.63)
Overreactivity C	2.60 (0.89)	2.60 (1.12)	2.33 (0.97)	2.40 (0.98)	2.88 (1.05)	2.85 (1.04)	2.63 (1.07)	2.60 (1.08)	2.88 (1.08)	2.76 (1.05)	2.61 (1.05)	2.45 (0.87)

M = mother reported, T = teacher reported, C = child reported

Pre = pretest (T1), Post = posttest (T2), Fu1 = follow-up 1 (T3), Fu2 = follow-up 2 (T4)

**Table 2:** Fit statistics for final univariate LGMs, bivariate LGMs, and cross-lagged path models

Model	χ*2 (df)*	*p*	CFI	RMSEA
*Univariate LGMs*				
Reactive aggression M^a^	15.94 (14)	0.317	0.993	0.039
Proactive aggression M	8.80 (15)	0.888	1.000	0.000
Reactive aggression T^b^	21.98 (14)	0.079	0.936	0.080
Proactive aggression T	21.47 (15)	0.123	0.963	0.070
Positive parenting C	10.46 (15)	0.790	1.000	0.000
Overreactivity C	9.31 (15)	0.861	1.000	0.000
*Multivariate LGMs*				
Positive parenting & reactive aggression M^a^	60.39 (71)	0.814	1.000	0.000
Positive parenting & proactive aggression M	57.55 (72)	0.892	1.000	0.000
Positive parenting & reactive aggression T^b^	79.28 (71)	0.234	0.982	0.036
Positive parenting & proactive aggression T	72.09 (72)	0.475	1.000	0.004
Overreactivity & reactive aggression M^a^	66.67 (71)	0.624	1.000	0.000
Overreactivity & proactive aggression M	58.45 (72)	0.875	1.000	0.000
Overreactivity & reactive aggression T^b^	92.42 (71)	0.045	0.934	0.058
Overreactivity & proactive aggression T	73.60 (72)	0.426	0.996	0.016
*Cross-lagged path models*				
Positive parenting & reactive aggression M	49.47 (48)	0.414	0.997	0.019
Positive parenting & proactive aggression M	47.10 (48)	0.501	1.000	0.000
Positive parenting & reactive aggression T	46.19 (42)	0.303	0.991	0.033
Positive parenting & proactive aggression T	59.87 (48)	0.117	0.976	0.053
Overreactivity & reactive aggression M	68.05 (54)	0.095	0.968	0.054
Overreactivity & proactive aggression M	45.90 (42)	0.314	0.988	0.032
Overreactivity & reactive aggression T	67.47 (42)	0.008	0.920	0.083
Overreactivity & proactive aggression T	54.89 (42)	0.088	0.964	0.059

M = mother reported, T = teacher reported, C = child reported

CFI: Comparative Fit Index; RMSEA: Root Mean Square Error of Approximation

^a^Revised model, slope factor loading of T2 is freely estimated

^b^Revised model, slope factor loading of T4 is freely estimated

In univariate LGMs, the intercept mean refers to the average initial level (fixed effect). Wald tests were used to compare intercept means in the child intervention condition, child + parent intervention and control condition. For all constructs, there were no significant differences in intercept means between the three groups. Thus, children showed similar levels of all six constructs at pretest, which indicates that randomization succeeded.

### Correlated Change

Fit statistics for all bivariate LGMs are included in Table 2 and slope-slope correlation coefficients are included in Table 3. All models showed good model fit (Table 2). Correlations between the slopes of aggression and the slopes of perceived parenting were non-significant in all three groups for most constructs (Table 3). However, there were significant correlations between the slopes of perceived overreactivity and the slopes of proactive aggression in the child + parent intervention condition. This was the case for both mother and teacher reported proactive aggression. Thus, in the child + parent intervention condition, there was correlated change between perceived overreactivity and (mother and teacher reported) proactive aggression, indicating that if proactive aggression decreased, perceived overreactivity decreased as well.

**Table 3:** Correlated change: parameter estimates from bivariate Latent Growth Models

Model	Child intervention			Child + Parent intervention			Control		
	*r*_ss_	*SE*	*p*	*r*_ss_	*SE*	*p*	*r*_ss_	*SE*	*p*
1. Positive parenting & reactive aggression M	0.04	0.02	0.138	0.02	0.04	0.623	0.04	0.04	0.353
2. Positive parenting & proactive aggression M	-0.01	0.02	0.453	0.00	0.02	0.923	0.01	0.03	0.783
3. Positive parenting & reactive aggression T	0.10	0.05	0.841	-0.06	0.07	0.409	0.01	0.06	0.916
4. Positive parenting & proactive aggression T	0.02	0.03	0.481	-0.01	0.04	0.777	0.06	0.04	0.111
5. Overreactivity & reactive aggression M	0.05	0.06	0.448	0.01	0.07	0.863	0.02	0.05	0.769
6. Overreactivity & proactive aggression M	0.02	0.03	0.412	0.06	0.04	0.049	-0.01	0.04	0.763
7. Overreactivity & reactive aggression T	0.13	0.09	0.167	0.21	0.12	0.078	-0.07	0.08	0.378
8. Overreactivity & proactive aggression T	-0.01	0.05	0.903	0.10	0.05	0.036	-0.01	0.07	0.842

M = mother reported, T = teacher reported, *r*_ss_ = slope-slope correlation

### Cross-Lagged Models

Model fit statistics of the final cross-lagged models are summarized in Table 2 and parameter estimates in Table 4. For all models except Model 3 (positive parenting and teacher reported reactive aggression), constraining the stability paths of aggressive child behavior did not result in a significant decrease in model fit. Thus, the stability paths of reactive and proactive aggression were time invariant for these models. Constraining the stability paths of perceived parenting resulted in a significant decrease in model fit for all models except Model 2 (positive parenting and mother reported proactive aggression) and Model 5 (overreactivity and mother reported proactive aggression). This indicates that the stability of parenting changed over time. Most stability paths of aggressive behavior and positive parenting were significant (see Figs. 2, 3, 4, and 5 for exceptions). For all models, constraining the cross-lagged paths did not result in a significant decrease in model fit. Thus, the cross-lagged paths from aggressive behavior to perceived parenting and from perceived parenting to aggressive behavior appeared to be time invariant.

**Table 4:** Parameter estimates from cross-lagged path models

Model	Child intervention			Child + Parent intervention			Control condition		
	*B*	*SE*	*p*	*B*	*SE*	*p*	*B*	*SE*	*p*
1. Positive parenting & reactive aggression M									
Aggression → Parenting	-0.08	0.04	0.056	-0.03	0.04	0.527	-0.05	0.05	0.360
Parenting → Aggression	-0.07	0.06	0.156	-0.09	0.08	0.281	-0.08	0.07	0.289
2. Positive parenting & proactive aggression M									
Aggression → Parenting	-0.13	0.06	0.031	-0.10	0.06	0.082	-0.12	0.07	0.075
Parenting → Aggression	-0.00	0.04	0.940	-0.04	0.05	0.413	-0.01	0.05	0.920
3. Positive parenting & reactive aggression T									
Aggression → Parenting	-0.02	0.03	0.534	-0.04	-0.04	0.315	0.01	0.04	0.783
Parenting → Aggression	0.05	0.07	0.453	0.05	0.06	0.447	0.05	0.06	0.447
4. Positive parenting & proactive aggression T									
Aggression → Parenting	-0.02	0.04	0.664	-0.02	0.03	0.558	0.02	0.05	0.724
Parenting → Aggression	0.07	0.07	0.310	-0.02	0.07	0.746	0.15	0.09	0.088
5. Overreactivity & reactive aggression M									
Aggression → Parenting	0.08	0.08	0.345	0.16	0.07	0.035	0.00	0.07	0.950
Parenting → Aggression	0.07	0.05	0.298	0.05	0.05	0.326	0.02	0.04	0.578
6. Overreactivity & proactive aggression M									
Aggression → Parenting	0.18	0.09	0.036	0.20	0.10	0.035	0.14	0.10	0.177
Parenting → Aggression	0.01	0.03	0.880	0.10	0.03	0.773	-0.03	0.03	0.383
7. Overreactivity & reactive aggression									
Aggression → Parenting	-0.01	0.07	0.858	-0.05	0.06	0.330	-0.09	0.07	0.155
Parenting → Aggression	0.05	0.07	0.539	0.03	0.06	0.673	0.08	0.07	0.232
8. Overreactivity & proactive aggression									
Aggression → Parenting	-0.01	0.06	0.801	-0.02	0.06	0.748	-0.12	0.06	0.063
Parenting → Aggression	0.06	0.06	0.285	0.00	0.06	0.991	0.07	0.06	0.300

M = mother reported, T = teacher reported

Parameter estimates for the final cross-lagged models are included in Table 4.^4^ Standardized parameter estimates are displayed in Figs. 2, 3, 4, and 5. In three of the eight cross-lagged models, significant child-driven effects from aggressive behavior to perceived parenting were found. Specifically, cross-lagged paths from mother reported proactive aggression to perceived positive parenting were significant in the child intervention condition (Fig. 3). Cross-lagged paths from mother reported reactive aggression to perceived overreactivity were significant in the child + parent intervention condition (Fig. 4). Moreover, Wald tests showed that this cross-lagged path was significantly stronger in the child + parent intervention condition than in the control condition (Wald = 4.66, *p* = 0.031). Cross-lagged paths from mother reported proactive aggression to perceived overreactivity were significant in both the child and the child + parent intervention conditions (Fig. 5). Thus, child-driven effects were found for mother reported aggression, but not for teacher reported aggression. The cross-lagged path from mother reported reactive aggression to perceived positive parenting approached significance (*p* = 0.056) in the child intervention condition (Fig. 2).

## Discussion

The aim of this study was to compare the associations of child and parent behaviors between developmental and intervention contexts. Specifically, we examined whether the association between (changes in) parenting and (changes in) aggression over time differed in three conditions: a child intervention condition, a child + parent intervention condition and a control condition. There was no evidence of bidirectionality between aggressive child behavior and perceived parenting in the normative developmental (control) context: changes in aggression and changes in perceived parenting were not correlated, and aggression and perceived parenting were not related above and beyond the stability of both behaviors. However, in the intervention contexts, the trajectories of change in aggressive child behavior and change in parenting were related. Specifically, decreases in aggressive child behavior were related to increases in perceived positive parenting and decreases in perceived overreactivity.

The absence of associations between aggression and perceived parenting in the control condition (a general developmental context) is consistent with earlier research on bidirectional influences in a general developmental context (Vuchinich et al. 1992). However, these results are in contrast to studies that report child-driven, parent-driven or bidirectional effects (e.g., Huh et al. 2006; Larsson et al. 2008; Pardini et al. 2008). A possible explanation for the absence of an association between aggressive child behavior and perceived parenting in the control condition can be derived from the characteristics of this sample. In the current study, children in the control condition showed elevated levels of aggressive behavior. Vuchinich et al. (1992) included a community sample of at-risk adolescents who also showed elevated levels of aggression and also reported the absence of bidirectional relations in a general developmental context. However, other studies on bidirectional associations mainly included community samples, without elevated levels of aggression (Huh et al. 2006; Larsson et al. 2008; Pardini et al. 2008). It might be possible that in the current study, parents of children in the control condition became discouraged as their attempts to control their child’s aggression failed and as a result tended to withdraw from interactions with their child and gave up on addressing aggressive behavior. This explanation is consistent with findings showing that high child problem behavior (McBride et al. 2002) and low parental sense of competence (Coleman and Karraker 1998) are related to parental involvement. However, it is also possible that these parents never tried these parenting styles. Another explanation is that the statistical power to detect these effects was too low because bidirectional associations are typically small in magnitude. Studies with larger sample sizes are more likely to detect these effects (Cohen 1992).

There was more evidence for associations over time between parenting and aggression in the intervention contexts. Trajectories of change in parenting and aggression were related in the intervention conditions, but not in the control condition. In addition, cross-lagged models showed that in the child intervention condition, aggressive behavior (reported by the mother) affected perceived parenting over time. In the child + parent intervention condition, where parenting skills were directly targeted, there was also more evidence for child-driven than for parent-driven effects. Thus, in both intervention conditions, aggression (reported by the mother) had a greater impact on parenting (reported by the child) than parenting had on aggression. These child-driven effects were found for both positive and negative parenting.

In both the child and child + parent intervention conditions, parents were aware that their child received an intervention for aggressive behavior. In addition, parents of children in the child + parent intervention condition received an intervention themselves. Earlier research has shown that parents who followed the additional parent intervention were more involved than parents who did not follow it (Stoltz et al. 2015). It is possible that this awareness and increased involvement made parents more responsive to their children, which facilitated their positive parenting behavior and reduced their negative, overreactive parenting behavior. Moreover, the intervention may have increased parents’ sense of competence (Deković et al. 2010). Parents who feel more competent have been found to react more warmly and responsively and less hostile and inconsistently to their child (Gondoli and Silverberg 1997; Sanders and Woolley 2005).

It should be noted that child-driven effects were found in mother-reported aggression models but not in teacher reported models. Pardini et al. (2008) also found that the influence of child behavior on changes in parenting was stronger when child conduct problems were measured by parents rather than teachers. Whereas in Pardini et al. (2008) this finding could be attributed to shared method variance because parents reported on both conduct problems and parenting, this was not the case in our study. Thus, it can be concluded that child-driven effects might be stronger when parents, instead of teachers, report on children’s aggression. An explanation for this reporter difference may be that children’s aggression at home is more likely to influence parenting than their aggression at school. An alternative explanation is that parents of children in the intervention groups simply rated the aggressive behavior of their child differently because they knew that their child had received an intervention.

Due to the fact that different types of aggression (reactive/proactive) and parenting (positive parenting/ overreactive parenting) were analyzed with separate models, it was not possible to statistically test differences between them. Moreover, differences did not appear to be consistent across analyses. Specifically, the bivariate LGM method appeared to show more evidence for correlated change between the slopes of proactive aggression and overreactive parenting than between other constructs whereas the cross-lagged path modelling method showed significant child-driven effects for both proactive/reactive aggression and positive/overreactive parenting. Although more significant child-driven effects appeared to be present in proactive aggression and overreactive parenting models, these differences could not be tested. So, although tentative, statistical reasons do not justify a conclusion that the association between (changes in) proactive aggression and (changes in) overreactivity is stronger than between other constructs. It is interesting for future research to focus on the possible difference between type of aggression and type of parenting with regard to (bidirectional) associations in an intervention context.

The findings of this study are partly consistent with the results of Shaffer et al. (2013) who reported that bidirectional effects were smaller than the temporal stability of parenting and child behavior in an intervention context. In the current study however, some evidence was found for child-driven effects from perceived parenting to aggressive child behavior in the intervention conditions. These effects were not found in the study of Shaffer et al. (2013). The intervention that participants in the study of Shaffer et al. (2013) followed was a multi-modal child and parent intervention that consisted of seven brief treatment modules. So, whereas in the current study, parents followed a specific intervention that was comparable to the child intervention, participants in the study of Shaffer et al. (2013) followed a broad intervention. It is possible that in the current study, parents were more involved with the intervention, and therefore, were more responsive to their child’s problem behavior.

### Strengths and Limitations

This study has both strengths and limitations. Strengths were: the inclusion of three conditions, which enabled us to test whether bidirectional associations differ in an intervention context versus a general developmental context; multiple reporters of aggression; child reports of perceived parenting; the assessment of both negative and positive parenting; and the use of different analyses to examine bidirectionality.

A limitation of the current study was the relatively small sample size. Although we share this shortcoming with many other intervention studies (e.g., Weisz et al. 2005), this might have diminished the power to detect effects. Sample size is crucial in the estimation and interpretation of SEM/LGM results because it is used to estimate standard errors and parameter estimates. The more variables and paths are included in a model, the larger the sample size needs to be (Stull 2008). However, some research suggests that LGM has more statistical power to detect group differences in growth trajectories than traditional methods, such as repeated measures ANOVA (Fan 2003). Monte Carlo simulations have demonstrated that basic LGMs hold up quite well with relatively small samples (Muthén and Muthén 2012). Thus, LGM may not require a very large sample to have sufficient power.

Another methodological limitation of this study was the dropout rate of parents who followed the parent intervention. We choose to analyze data as recommended, with an intention-to treat analysis (White et al. 2011). However, the dropout rate may have influenced the possibility to detect differences in bidirectional associations between the child intervention and the child + parent intervention conditions. Moreover, the present study relied on child reports of perceived positive/negative parenting. Although Gonzales et al. (1996) found that child reports had the highest correspondence with independent ratings of parenting, more confidence could have been placed in the results if findings of the present study were replicated using direct observations of parenting.

Notwithstanding these limitations, this study contributed to the bidirectional relationships literature by examining whether associations between aggression and parenting are different in an intervention context compared to a general developmental context. The use of bivariate latent growth curve analyses enabled us to examine correlated change over the period from pretest to one-year after intervention termination. And with cross-lagged path analyses, the direction and timing of effects could be examined. Results showed that associations between aggressive child behavior and parenting are different in an intervention context compared to a general developmental context. Specifically, aggressive behavior and perceived parenting were unrelated over time for children with elevated levels of aggression who did not receive an intervention. Whereas, in an intervention context, decreases in children’s aggression were related to increases in perceived positive parenting and decreases in perceived overreactivity. The findings of this study shed new light on bidirectional processes and contribute to a better understanding of intervention processes. Although our findings should be replicated, they underscore the importance of child-driven processes in interventions aimed at children, but also in interventions aimed at both children and their parents.

## Footnotes

1 There are fewer participants in the control condition because we used a sampling method in which 2/3rd participated in the intervention conditions (child intervention condition and child + parent intervention condition) and 1/3rd in the control condition.

2 Additional stability paths and residual correlations are not depicted in Figs. 2, 3, 4, and 5, in order to reduce the complexity of the figures. This information is available on request of the first author.

3 To conserve space, model fit statistics are only reported for final models. Fit statistics and results of Chi-square difference tests are available on request of the first author.

4 To conserve space, parameters estimates are only reported for cross-lagged paths. Additional parameters are available on request of the first author.
